# Exploring the Links between Ethnobotany, Local Therapeutic Practices, and Protected Areas in Santa Catarina Coastline, Brazil

**DOI:** 10.1155/2012/563570

**Published:** 2011-11-17

**Authors:** Sofia Zank, Natalia Hanazaki

**Affiliations:** Department of Ecology and Zoology, Federal University of Santa Catarina, Campus Trindade, 88010-970 Florianópolis, SC, Brazil

## Abstract

We investigated the knowledge of medicinal plants in two areas proposed for the creation of protected areas for sustainable use in the city of Imbituba (SC). In this study, we analyzed the influence of gender, form of learning, and modern medicine on medicinal plant knowledge while also reflecting on the relationship of this knowledge to *in situ* conservation. Data collection was conducted through structured interviews, free listings, guided tours, and collection of botanical material. 197 species of medicinal plants belonging to 70 botanical families were recorded. Gender and the form of learning were factors that significantly influenced the similarity of the knowledge of medicinal plants among the informants. We also observed the existence of a therapeutic pluralism among key informants. Local medicinal plant knowledge emphasizes the importance of strategies to create protected areas of sustainable use as a way to ensure the maintenance of traditional lifestyles and associated local knowledge.

## 1. Introduction

Among the known natural resources managed by human populations, medicinal plants stand out as important links between people and the natural environment, a knowledge that is present in many local communities and with a large abundance of known and used species [1–6]. The knowledge of medicinal plants in traditional communities is closely linked to the practical aspect (doing), having been built over the years by social interactions of people among themselves and with the surrounding environment, this knowledge assumes an important role in the identity formation and self-recognition of these populations.

The use of medicinal plant in therapies is a widespread practice in folk medicine [4, 7, 8]. Access to modern medicine by the local population does not eliminate the use of local medicinal practices, which is often included in therapeutic pluralism of the communities. While not eliminating the practice of folk medicine, the introduction of modern medicine may lead to the disappearance or modification of some traditional practices [7, 9]. Other factors may also influence the knowledge of medicinal plants, such as gender, form of learning, religion, and age among others. In relation to gender, for example, several studies show that different occupations between men and women end up influencing their knowledge of plants [10–13].

Local knowledge can also be influenced by changes in traditional practices. The loss of natural areas, due to urbanization or large-scale agriculture, could influence significantly traditional practices. Natural areas are a source of therapeutic resource for many communities, and also a space for social organization and cultural reproduction. In the coastal region of Brazil, uncontrolled urban expansion and property speculation have led to extensive loss of natural areas, culture, and traditions of communities living in these areas [5, 8]. The access to territory is of primary importance to maintain the local and traditional way of life, because the environment of each given local community has the conditions for their cultural reproduction and identity [14]. 

As a form of resistance to the urbanization pressure, some traditional communities have been organizing and seeking recognition of their rights of access to land and natural resources. A strategy for recognition of their rights is the establishment of protected areas for sustainable use, allowing the maintenance of traditional livelihoods, sustainable use, and conservation of plant resources [15]. The latter reality can be seen in the south-central coast of Santa Catarina, where local communities have requested the creation of two protected areas (PAs) for sustainable use, an Extractive Reserve (RESEX) for the Artisanal Fisheries of Imbituba and Garopaba and the Areais da Ribanceira Sustainable Development Reserve (RDS). The establishment of these PAs is a form of withstanding pressures and ensuring access to territory and natural resources for local communities.

Studies of how local knowledge is organized and influenced are important for understanding the processes and maintenance of local knowledge generation. The preservation of cultural identity requires that local knowledge is passed from generation to generation [16], and that the processes of knowledge generation are maintained. Moreover, these studies collaborated to incorporate the difference in knowledge of native plant into strategies for conservation.

In this context, this study aimed to investigate the knowledge about medicinal plants in two regions proposed for protected areas for sustainable use in the municipality of Imbituba (SC). As well as seeking to analyze the influence of gender, form of learning, and modern medicine on medicinal plant knowledge. In this study, reflections are made on the relationship of medicinal plant knowledge with the maintenance of traditional livelihoods and biodiversity conservation.

## 2. Area of Study

**Figure 1:**
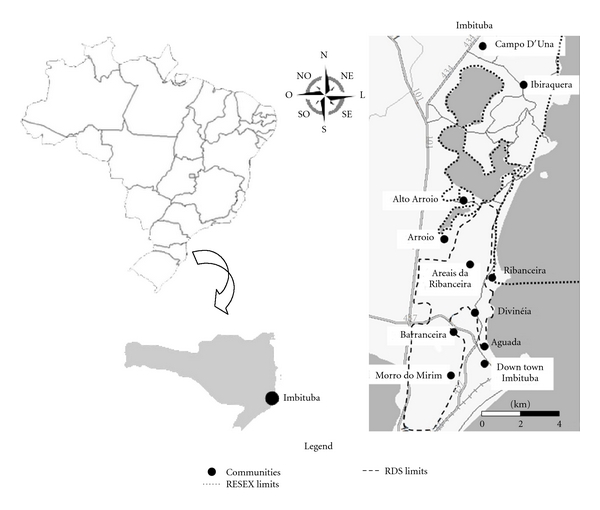
Map of study area showing the researched communities and the proposed boundaries of the protected areas in the municipalities of Imbituba, Santa Catarina (Brazil).

The municipality of Imbituba is located on the south-central coast of the state of Santa Catarina (Brazil), about 90 km south of the capital Florianópolis (Figure 1). Imbituba is a port city, with a population of about 40,000 inhabitants. All municipality is considered urban, and this means that people who are farmers have easy access to market, hospital, and other modern facilities.

The coastal landscapes present in Imbituba are heterogeneous and complex spatial structures [17, 18]. Imbituba is located in the Atlantic Forest biome, where a mosaic of different ecosystems are present, ranging from *restinga* to dense ombrophyllous forest. Other features of this landscape include lagoons, swamps, wooded *restingas*, grassy *restingas*, shrub *restingas*, *butiazais* (areas with high densities of an endemic small palm, *Butia catarinensis *Noblick & Lorenzi), and dense submontane ombrophyllous forest [17, 18].

The *restinga* vegetation is present in sand dune ranges composed mostly of endemic vegetation, which includes “originally herbaceous formations, undergrowth, shrub, or tree, which can occur in mosaics and also have areas that are naturally devoid of vegetation; such formations may have been kept as primary or transformed into secondary, as a result of natural processes or human intervention” [19]. 

The occupation of the region is long standing, formed in 1715 as the core of Azorean colonization and pioneers. Until the 1960s, families ensured their livelihoods with a combination of agriculture, fishing, and hunting [20]. The agricultural management made use of slash-and-burn farming, consisting of the accumulation of branches that were incinerated at the same time to clear and fertilize croplands [18].

The production system connected to family farming and artisanal fishing remained until the late 1970s, when the increase of tourist activities, with the implementation of the BR-101 and the intense property speculation, strongly contributed to a distortion of the traditional populations [17, 20]. At this time the Imbituba Industrial Complex was implemented in the Areais da Ribanceira region with the promise of creating new jobs that did not materialize. Thus, many farming families were displaced, but continued to occupy the area and practice agriculture [17].

Farmers and traditional fisherman in Imbituba have been going through an intense process of progressive land loss in order to carry out their way of life, such as access to the sea, lakes, agricultural fields, and the resources from these areas [20]. As a way to resist these pressures, farmers and fishermen in Imbituba proposed the creation of two PAs, an RESEX and an RDS. The purpose of this PA is to protect natural environments and to ensure the maintenance of the farmers and fishermen's livelihoods [17, 18]. Besides these two PAs in the making, the region is covered by the Environmental Protection Area (APA) of the southern right whale, founded in 2000, in order to protect the southern right whale (*Eubalaena australis, *Desmoulins, 1822) and ensure the sustainable use of natural resources in the region.

The initiative for the creation of the PAs comes from local community organizations and was supported by different groups. The process of creating the RESEX began in 2005, on request of the Forum Agenda 21 of Ibiraquera and the Association of Fishermen of Ibiraquera (ASPECI). This PA includes the municipalities of Imbituba and Garopaba, with an area of approximately 19.930 hectares, covering the lagoons of Ibiraquera, Doce, Encantada and Garopaba, and the adjacent coastline. The most significant portions are covered by water sheets (sea and lakes) and the area of the extractive reserve falls partly within the limits of the of the southern right whale protected area [17].

The request for the creation of RDS Areais da Ribanceira was presented by the Rural Community Association of Imbituba (ACORDI) in August 2005. The area proposed for RDS covers and encompasses agricultural areas, *restinga* ecosystems, and dense ombrophyllous forest. These environments are also used for the extraction of plant resources such as medicinal plants and *B. catarinensis*. The total area proposed for the RDS is approximately 2.100 hectares, and part of the area is included in the southern right whale protected area [18].

The procedures for the creation of RESEX are in an advanced stage of negotiations, only requiring the final approval by the Brazilian Ministry of Environment. However, there are still several steps to be accomplished in the procedures for the RDS creation.

## 3. Methods

### 3.1. Data Collection

The ethnobotanical information on medicinal plants was collected during the period between August 2009 and June 2010, through structured interviews with key informants, free lists, field notes, and guided tours [21]. The participation of informants was dependent on the acceptance of the term of prior informed consent (TAP). 

Data was collected in 11 localities of Imbituba: Aguada, Areais da Ribanceira, Arroio, Alto Arroio, Barranceira, Campo D'Una, Imbituba Center, Divinéia, Ibiraquera, Morro do Mirim, and Ribanceira. These localities, or neighborhoods, are close to each other and with easy access, so people who live in a certain locality have relationships with people of other localities.

Sampling of study subjects was intentional; interviews were conducted with key informants, also called local experts, were recognized as having a specific knowledge. The selection of informants was based on the “snowball” method [22], in which each informant indicates other informants to cover the largest number of people who have the specific knowledge being investigated. The following were criteria for informant inclusion: adults, residents for over 20 years in the region and had knowledge of medicinal plants. Sampling was initiated through the indication of community leaders and researchers who developed studies in the communities and ended when there were no more new indications. Some informants were included randomly by accident, while looking up information on the homes of other key informants. The interviews were structured [21] and based on a preset of questions regarding the socioeconomic status of the informants, the way of learning about medicinal plants, differences in present and past knowledge and use of medicinal plants, traditional therapies, modern medicine, and a free list of known medicinal plant species.

A pilot study was conducted with three people to verify the need to adjust the methodology [21]. The interviews in the pilot study were included in the data, since the questionnaire underwent only minor modifications. 

The free-list method, in which participants are asked to list the plants they know [21], was conducted with all informants and was intended to raise the species richness of known medicinal plants and specific information about these plants (the use/purpose, how it was obtained, and collection sites). The plants mentioned were collected in guided tours. The tour was held after the interview, taking place in the backyard of the respondent's home. Tours were also held in areas of native vegetation with informants who cited wild plants and those that were available for such an activity. 

The collection of cited plant samples was conducted following the standard procedure for ethnobotanical species collection [21]. Plant materials were identified by specific bibliographies and consultations with experts. Plant material was deposited in the herbarium FLOR (UFSC/SC) and in the collection of the Human Ecology and Ethnobotany Laboratory/UFSC. Identification followed the classification system of APG II and scientific names were checked by consulting the website of the Missouri Botanical Garden [23].

Some mentioned plants were not collected due to their absence in the vicinity of homes, low abundance of some native species in the natural ecosystems, and walks with elderly informants that could not be carried out. The plants that were not collected were identified according to the collected specimens that had the same common name, or if there were no collected specimens, plants were identified by the description and by the common names. The specimens with common names that include more than one scientific species (e.g., *espinheira-santa*, *anador*, *quina*) or that there is no reference in the literature were classified as unidentified and were excluded from the analysis.

In some situations, informants were visited more than once, in order to collect plant specimens. Any additional plants that arose during these visits were not included in the comparative analysis between the informants, so that the difference in sampling did not influence the results. 

The return of the results from the study occurred during the research, according to the demands presented by the community. Technical reports were prepared to assist in the legal process of access to land, lectures were held at community events and a workshop to return study results. An illustrative brochure publicizing the local ecological knowledge was also developed.

### 3.2. Data Analysis

Interviews and free lists were analyzed using descriptive statistics. The classification of indicated therapies was done according to World Health Organization (WHO) [24], yet other categories were added because the community recognize some local diseases that were not classified by WHO. To analyze known medicinal species a list of mentioned plants was prepared, with the plants common name/ethnospecies (in this study, ethnospecies was considered a synonym of common name, i.e., the identification of plants is done from the knowledge of the interviewees), botanical classification and frequency of citation. Randomized species-accumulation curve was used, seeking to assess the expected richness of used and known plants by the number of plant species [25]. This analysis was performed using the program EstimateS version 8.0 [26] with the Chao 2 richness estimator. 

To analyze the influence of gender (male and female) and forms of learning (by elderly and courses/books) on knowledge of medicinal plants, the species richness for each group was compared using a *t*-test for gender and Mann-Whitney *U*, for form of learning—because the data did not show normality and homogeneity. The composition of the species mentioned by each group was compared using the ANOSIM analysis, using a matrix of presence and absence of cited species, where the informants were the sampling units and species mentioned were the variables. In this matrix, species mentioned by only one informant were excluded. From the absence/presence matrix, the Sorensen similarity matrix was calculated using the clustering method UPGMA. This analysis was performed using the program Primer 6.0 Beta [27]. The influence of form of learning was also analyzed through frequency of information about the question of how the person have learned about medicinal plants.

The influence of modern medicine was analyzed through the frequency of the medicinal plants and manufactured drugs that have been used by the family in the last month. Frequency analysis also was done for the use of doctors/agent of popular medicine and the perception of change on medicinal plants knowledge.

## 4. Results and Discussion

### 4.1. Interviews

Twenty-three key informants, 9 men and 14 women, were interviewed. It is noteworthy that in three interviews with male informants their wives were also present. Nine participants are members of ACORDI (Rural Community Association of Imbituba) and are involved in the process of creating the RDS. Five informants, or people of their households, are involved in the movement to create the RESEX.

The informants were between the ages of 40 and 86 years, the average being 68.5 years (SD 9.5). Fourteen are married, seven widowed, and two single. The families of the respondents have an average of 4 children (ranging from 0 to 9), living an average of 4 persons per household (ranging from 1 to 7). In regards to income, 65% are retired, 9% receive a pension, and 8% have income from fishing and agriculture, and 8% have their income from other services (health sector and school). Some retired people have been employed on past, but they maintain farm practices during all live, getting more expressive during retiring time.

### 4.2. Knowledge of Medicinal Plants

Through interviews and guided tours 218 ethnospecies of medicinal plants were recorded, of which 197 were identified taxonomically, belonging to 70 botanical families (Table 1). The families Asteraceae (16%) and Lamiaceae (8.5%) amounted to the highest number of species of cited medicinal plants. Asteraceae and Lamiaceae are among the families with the largest number of medicinal species cited in areas of *restinga* [2, 7, 21, 23].

This study showed a higher species richness compared with other ethnobotanical medicinal plant surveys conducted in the coastal regions of Brazil [8, 13, 28, 29]. During a study in Sertão do Peri (Florianópolis, SC), 114 species of medicinal plants were found, through 13 interviews, where all households of the site were visited, with refusal of participation by some informants [29]. For the region of Itapoá (SC), 109 species were recorded, resulting in 90 interviews in which informants were selected through random sampling [13]. In a study conducted with 14 key informants in a *caiçara* community in Vila Velha (ES), 86 species were recorded [28]. In Pinto et al. [8] 98 species of medicinal plants were reported in Itacare (BA), by 26 informants, selected by nonrandom sampling. It is worth noting that these studies used different methods for ethnobotanical survey of medicinal plants, which can influence the values of richness, so the comparison between species richness should be done with caution. 

**Figure 2:**
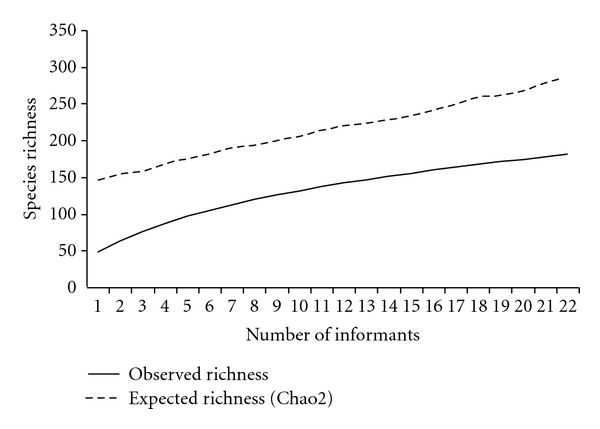
Accumulation curve and estimation of richness of known medicinal plants in the municipality of Imbituba, with a richness of 197 observed species cited by 23 key informants.

The richness estimator Chao 2 estimated 286 species for the region studied (Figure 2); therefore, over 89 more medicinal plant species are expected to be found in the region than were sampled. 

**Figure 3:**
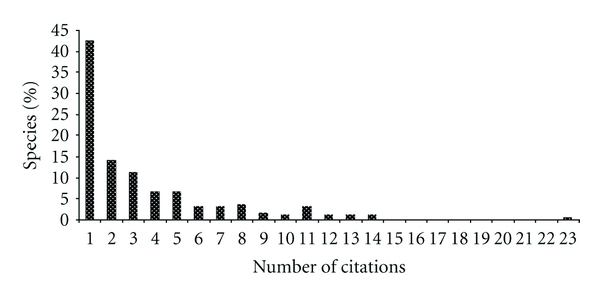
Percentage of medicinal plants species (*n* = 197) according to the number of times they were cited by 23 key informants in the municipality of Imbituba, SC.

When the number of citations of each species was measured, it was observed that 43% of the species were cited by only one informant (Figure 3), which demonstrates that there is a significant percentage of knowledge that is not shared between the local experts. In addition, the high number of rare species, cited by only one or two informants, influences the expected value of richness, which was calculated using the Chao 2 estimator, explaining 31% difference between the observed and expected richness (Figure 2). 

The species most often cited was *menta (Mentha* sp1.), cited by all informants. *Laranja* (*Citrus sinensis* (L.) Osbeck) and *menstruz* (*Coronopus didymus* (L.) Sm) were mentioned by 61% of informants. *Camomila* (*Chamomilla recutita* (L.) Rauschert) and *melissa (Lippia alba* (Mill.) N.E. Br. ex Britton & P. Wilson) were cited by 57% of the informants.

These species also appear as the most cited in other studies. In Giraldi and Hanazaki [29], *menta* (*Mentha* sp.), *camomila* (*Chamomilla recutita* (L.) Rauschert), and *laranja* (*Citrus sinensis* (L.) Osbeck) also appeared as the most cited. In Albertasse et al. [28] and Merétika et al. [13], *menta* (*Mentha* sp.) was also one of the most cited species. In Pinto et al. [8], the most cited plants were *menstruz* (*Chenopodium ambrosioides* L.) and *erva-cidreira* (*Lippia alba* (Mill) N.E. Br.). It should be noted that the two most cited species are common, generally cultivated in backyards and gardens, with the exception of *menstruz*, but this plant is spontaneous and easily accessible.

**Figure 4:**
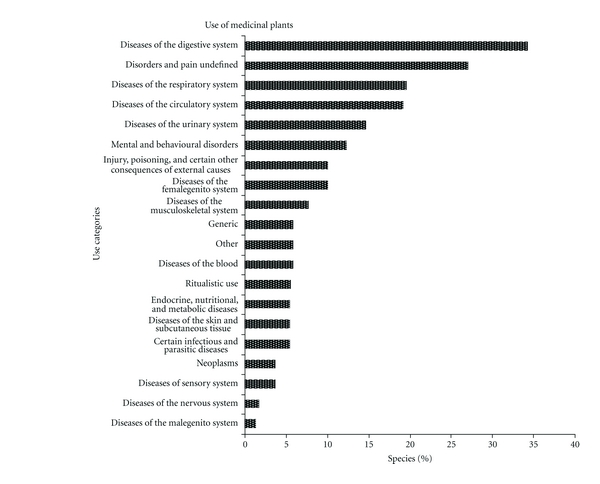
Percentage of medicinal plant species (*n* = 197) cited by 23 key informants in the municipality of Imbituba in relation to its therapeutic use category.

In relation to therapeutic uses, 18 categories were identified according to the body system they are used to treat (Figure 4). In addition to these categories, an “other” category was also included for diseases that do not fit any classification and the category “general,” for plants that were cited to treat any condition. Some plants were included in ritualistic category due to its manner of use. Plants were considered as ritualistic if used to treat the “evil eye” in order to give a “shower of protection,” to bless, among other uses. The main categories of use were digestive disorders (34%), undefined pain or conditions (19%), respiratory disorders (17%), and circulatory disorders (17%). Ethnobotanical studies conducted in other regions also found that digestive and respiratory system categories were cited as the main uses for medicinal plants [8, 12, 13, 28, 29].

**Figure 5:**
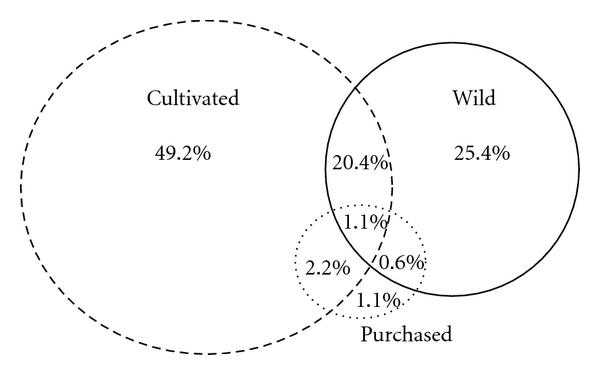
Percentage of medicinal plants species (*n* = 197) cited by 23 key informants from Imbituba, according to way of obtaining.

When informants were asked about how they obtain each medicinal plant—cultivated, wild, or purchased—it was found that most plants are grown in backyards and gardens (60%), however, not necessarily by the informants. A significant percentage of the used medicinal plants (36.5%) are considered wild and extracted from the surrounding environments. The types of collection environments ranged from sand dunes, forest (*restinga* and hillside), secondary forests, swamps, fields, and plants that grow spontaneously in fields and near the houses. A small percentage (3.5%) of the plants is bought by informants (Figure 5). The use of a significant number of wild plants, which are extracted from the surrounding environment, demonstrates the connection of the population with the environment and emphasizes the importance of preserving this knowledge so these practices may continue. As pointed out by Cunha [30], the threat to local knowledge is not simply to the knowledge itself, but the conditions of production of knowledge.

### 4.3. Gender Influences on Knowledge

The analysis conducted to evaluate the influence of gender generated differentiated and complementary results. Women have cited more plants (average 31, SD 12.7) than men (average 26.8, SD 18.7). The comparison between the number of medicinal plant citations among the groups was not significant for gender (*P* = 0.53). On the other hand, when these groups were compared in terms of cited species composition, significant differences were found. In the analysis of similarity, ANOSIM, the differences between groups of men and women was significant (*P* < 0.05). The difference in knowledge between men and women was also addressed by Hanazaki et al. [12], Case et al. [11], Merétika et al. [13], and other studies. Hanazaki et al. [12] found differences in the number of medicinal plants citations among men and women in some *caiçara* communities on the coast of São Paulo, where men cited more plants than women. In Merétika et al. [13], it was observed that women knew more medicinal plants than men, but the difference was not significant. In a study conducted in the Manus Islands (New Guinea), Case et al. [11] found significant differences in the identification of names and uses of plants between men and women. They found that men knew more about plants, but in relation to medicinal plants no differences were found. The similarity analysis is a complement for the comparative analysis between groups. As this study shows the difference in knowledge does not necessarily arise in the number of plant species cited, but the quality of knowledge—people from different groups know different plant species.

### 4.4. The Influence of Form of Learning

When asked how they learned about medicinal plants, 65% said they learned through family members, 13% learned through other experienced people in the community (e.g., traditional healers), 43% attended medicinal plant courses (e.g., courses given by a religious health organization called *pastoral da saúde*), 9% learned through books, and 9% by personal experience with plants and nature. The high incidence of local experts who participated in medicinal plant courses is due to the fact that there is a unit of the *pastoral da saúde *(the *Pastoral da saúde* is a nonprofit, civic-religious society linked to he Catholic Church, officially established in 1986), in the center of the city, which administered some courses in the community.

To compare the difference of knowledge to do the form of transmission, we define two groups. People who learned through older people (transmission one to few) as opposed to courses/books (transmission one to many). People who learned through older people have cited more plants (average 30, SD 20.3) than people who learned through courses/books (average 28.3, SD 8.0). The comparison between the number of medicinal plant citations among the groups was not significant for form of learning (*P* = 0.60). On the other hand, when these groups were compared in terms of cited species composition, significant differences were found (*P* < 0.01).

The form of learning, or the way of transmission, can influence the knowledge of medicinal plants in Imbituba. Some studies have demonstrated that the transmission “one to many”, as course and others forms of training, increases the homogeneity inside a population. This process maybe has happened in Imbituba with the course of *Pastoral da Saúde*. However, the transmission of knowledge in courses is seen as efficient, and the innovation can occur with facility and speed [31]. 

### 4.5. Therapeutic Pluralism and Traditional Knowledge of Medicinal Plants

Imbituba population has easy access to modern medicine. There is a hospital on the center of city, and health post and pharmacy in almost all localities. All informants have access to modern medicine and use it, but there is variation in the frequency in which they seek this resource. Regarding the use of medicinal plants, 91% of respondents reported using medicinal plants in the last month, but 13% of them had difficulty remembering which plants were used. In addition to medicinal plants, other traditional therapeutic practices are used by respondents, like the demand for *benzedeiras* (traditional healers). The *benzedeiras* were cited as a therapeutic resource for 70% of respondents; however, only 30% of the informants used this resource in the past. It is noteworthy that two of the informants are recognized as *benzedeiras* and are very popular with people in the community and other regions. Both were more than 80 years old when they were interviewed, and one of them passed away in September 2010. 

Two other informants learned some *benzeduras *from older members of their families and use these therapies only with family. One of the informants was a herbal medicine man and had a shop in his home where he sold herbal potions to the community in the past. He currently no longer performs this role, due to legal and financial difficulties in maintaining the store.

The *pastoral da saúde *unit in Imbituba held courses in medicinal plants for the community and currently has study groups on medicinal plants. While this center may facilitate the maintenance of traditional therapies—as a process of use of medicinal plants—by the dissemination and appreciation of medicinal plants, the devaluation of some therapeutic practices may also occur, such as *benzedura*. This form of transmission can also homogenize the knowledge of medicinal plants, as we have seen on the influence of form of learning. 

**Figure 6:**
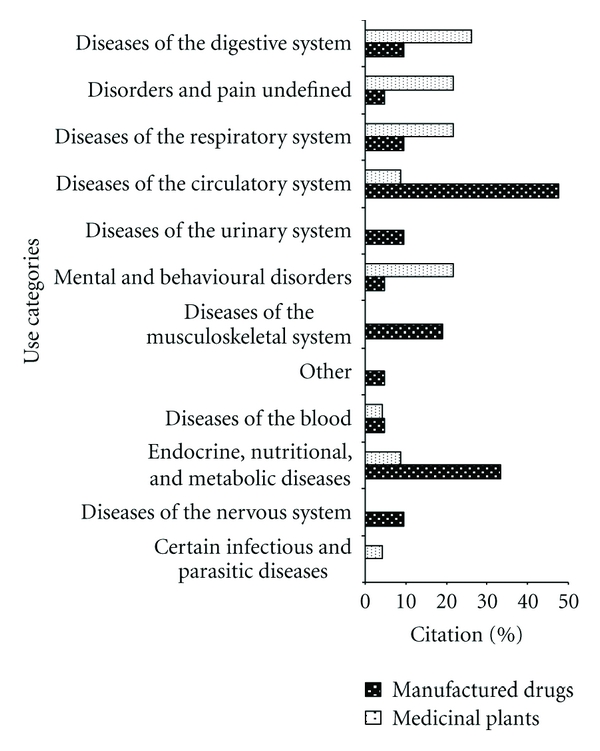
Percentage of types of diseases (*n* = 12) that are treated with manufactured drugs and medicinal plants by 23 key informants in the municipality of Imbituba.

Taking into consideration the manufactured drugs and medicinal plants used by informants in a month, there are perceivable differences in the types of illnesses that are treated by each of the therapeutic practices, and that they are used in a complementary way (Figure 6). Informants often use medicinal plants to treat diseases related to digestive disorders, pains, and undefined conditions, respiratory problems, and mental and behavioral disorders. On the other hand, manufactured drugs are preferred for treating circulatory, endocrine, nutritional, and metabolic diseases. 

Other studies that compared the use of medicinal plants and manufactured medicines also noted that medicinal plants are commonly used to treat diseases of the digestive and respiratory systems [7, 29], while manufactured drugs are used primarily to treat circulatory and endocrine systems [7, 29]. As discussed by Benítez et al. [32], medicinal plants are often used to treat simple ailments, that are not necessary to seek medical help, such as digestive problems and colds, especially, conditions that respond well to treatment with medicinal plants. 

When asked about changes in the use and knowledge of medicinal plants, 70% of respondents commented that the use of medicinal plants is a practice that has declined in relation to the past. On the other hand, 30% of respondents believe that the use of medicinal plants is increasing again, due to concerns about the negative effects of allopathic drugs and the influence of courses, such as the ones administered by the ministry of health.

“There's a difference. At that time there were no doctors, hospitals. Today it's just doctors and pills, they do not want to make herbal teas anymore.” (I7 ♀ Arroio).

“Today nobody believes. They want the herbal teas to heal in an instant. Today there are doctors and medicines for whatever condition in the pharmacy.” (I11 ♂ Imbituba center).

“Before, they did not use because they did not know the properties. Before it was not valued because it was not understood.” (I8 ♀ Arroio).

A therapeutic pluralism is perceived among local experts on medicinal plants, while people are using modern medicine and tradition practices in a complementary way (Figure 6). These data corroborate with Amorozo [7], who argues that folk medicine is influenced by modern medicine, it this does not destroy the existing systems, but adds to new possibilities. So illness can be seen as curable only by the doctor or by local experts, or people can treat the same disease through the two systems [33]. However, it is important to note that this survey was conducted only with local experts, who are known to have greater affinity to medicinal plants. Thus, it is important to also investigate how knowledge of medicinal plants and therapeutic pluralism are present in the community as a whole.

### 4.6. Traditional Knowledge and Sustainable Protected Areas

The data reflect the cultural importance of medicinal plants in Imbituba, even in the face of intense social, economic, and environmental changes that these local populations have been suffering. The maintenance of local knowledge encourages the conservation of natural ecosystems, in regards to the use of this resource, and strengthens the communities identity, helping to fight for their rights. 

The large number of medicinal species, that are considered wild by the local population, reflects the importance of surrounding environments for the maintenance and the production of this knowledge. In this context, the creation of the RESEX and RDS, which seek to ensure land and maintenance of livelihoods for local populations, will support the strengthening of their traditional practices, including those related to health and knowledge and use of medicinal plants. It is important to note that the designation of these populations as traditional should refer to their cultural and historical rights over the area [14], and thus enabling maintenance their of autonomy and capacity for change. The traditional population of Imbituba has assumed an attitude in favor of conservation as a political strategy, a fact that is observed in several traditional communities in Brazil. The creation of a sustainable use protected area has become one of the most common alternatives to ensure both the conservation and use of natural resources and the access to the territory [14].

Moreover, if the PAs are created, traditional knowledge will be important for the development of the management plan of the area, as well as the development of an use plan compatible with the cultural aspects and the demands of the community, including the differences of knowledge among groups and different interests that coexist within the local population. As discussed by Hanazaki et al. [34], if the management and the decision making process are conducted in a participatory way, local communities can become empowered and thus play important roles in the *in situ* conservation, incorporating local knowledge into management strategies.

## 5. Conclusion

The communities living in the vicinity of the two proposals for protected areas in the region of Imbituba have a significantly important knowledge of medicinal plants. The high proportion of known medicinal plants in this region reflects the importance that this therapeutic approach has within the social structure of these communities, even with the strong influence of urbanization and easy access to modern medicine. 

Gender and the form of learning are factors that significantly influence the similarity in knowledge of medicinal plants in the region of Imbituba. A therapeutic pluralism was identified in the region, where modern medicine and traditional practices are complementary to each other. There is a higher preference for one or the other depending on the type of the ailment. However, some informants perceive a devaluation of medicinal plants in relation to modern medicine by people in the community.

The richness of known medicinal plant species and the existence of traditional health practices demonstrate the resilience of traditional communities in the face of development pressures and urbanization that has been ongoing along the coast of Santa Catarina. This information is extremely important to the process of recognition and identification of these traditional populations and the fight for their rights through the creation of protected areas for sustainable use.

## Figures and Tables

**Table 1:** Medicinal plants (botanical classification, common name, and frequency of citation) cited by 23 key informants living in two areas proposed for protected areas of sustainable use in the municipality of Imbituba. No. is the number and collection: *F*: Herbarium FLOR (UFSC); *L*: Human Ecology and Ethnobotany (UFSC) lab collection; IC: identified in the field; and NI: not identified*¹*.

Botanical classification	Local name	Frequency of citation	No. collection
Adoxaceae			
*Sambucus australis* Cham. & Schltdl.	Sabugueiro	6	L1222
Alismataceae			
*Echinodorus grandiflorus* (Cham. & Schltdl.) Micheli	Chapéu-de-couro	4	L1139
Amaranthaceae			
*Alternanthera brasiliana* (L.) Kuntze	Meracilina, pinicilina	7	L1199
*Alternanthera *cf.* sessilis* (L.) R. Br. ex DC.	Anador	1	F38677
*Alternanthera dentata* (Moench) Stuchlik ex R.E. Fr.	Anador	1	L1114
*Alternanthera *sp1.	Gaiana	1	L1163
*Alternanthera* sp.	Anador	1	NI
*Beta vulgaris* L.	Beterraba	1	IC
*Chenopodium ambrosioides* L.	Erva-de-santa-luzia, erva-de-bicho	3	L1235
Amaryllidaceae			
*Allium sativum* L.	Alho	2	IC
Anacardiaceae			
*Mangifera indica* L.	Manga	1	L1188
*Schinus terebinthifolius* Raddi	Aroeira	1	IC
Apiaceae			
*Centella asiatica* (L.) Urb.	Pata-de-mula	1	L1205
*Foeniculum vulgare* Mill.	Funcho, endro	11	L1162
Apocynaceae			
*Asclepias curassavica* Griseb.	Erva-borboleta	3	L1149
*Catharanthus roseus* (L.) G. Don	Bambacá, figueira-inferno	1	F38679
*Hoya* sp.	Flor-de-cera	1	L1160
*Tabernaemontana catharinensis* A. DC.	Mata-olho	1	L1195
Araceae			
*Zantedeschia aethiopica* (L.) Spreng.	Copo-de-leite	1	IC
Arecaceae			
*Bactris lindmaniana* Drude	Tucum	1	NI
Aristolochiaceae			
*Aristolochia triangularis* Cham.	Cipó-mil-homens	12	L1143
Asparagaceae			
*Sansevieria trifasciata* Prain	Espada-de-são-jorge	1	IC
Asteraceae			
*Acanthospermum australe* (Loefl.) Kuntze	Féu-de-índio	1	L1158
*Achillea millefolium* L.	Mil-em-rama	2	IC
*Achyrocline satureioides* (Lam.) DC.	Marcela	7	L1192
*Arctium minus* Schkuhr	Bardana	1	L1120
*Artemisia absinthium* (Mill.) Y.R. Ling	Losna	4	L1183
*Artemisia alba* Turra	Cânfora, cânfora-da-horta	3	L1128
*Baccharis milleflora* DC.	Carqueja	1	L1130
*Baccharis* sp.	Carqueja	4	NI
*Baccharis trimera* (Less.) DC.	Carqueja	1	L1154
*Bidens pilosa* L.	Picão	11	L1209
*Calea serrata* Less.	Quebra-tudo	1	L1217
*Calea uniflora* Less.	Arnica	11	L1236
*Centratherum punctatum* Cass.	Saudade	1	L1225
*Chamomilla recutita* (L.) Rauschert	Maçanilha, camomila	13	L1184
*Cnicus benedictus* L.	Aratanga, caldo-santo, cardo-santo	8	L1131
*Cotula australis* (Sieber ex Spreng.) Hook. f.	Marcela-galega	8	L1193
*Cynara scolymus* L.	Alcachofra	3	NI
*Eupatorium inulifolium* Kunth	Erva-de-bicho, cambará-do-roxo	2	L1150
*Mikania cordifolia* (L. f.) Willd.	Guaco	1	L1168
*Mikania glomerata *Spreng.	Guaco	1	L1167
*Mikania laevigata* Sch. Bip. ex Baker	Guaco	5	L1237
*Mikania* sp1.	Guaco	1	L1238
*Mikania *sp.	Guaco	3	NI
*Pluchea sagittalis* (Lam.) Cabrera	Quitoco	1	L1218
*Polygonum acuminatum* Kunth	Erva-de-saracupa, Pimenta-d'água	1	F38676
*Solidago chilensis* Meyen		1	L1227
*Spilanthes acmella* Hutch. & Dalziel	Dormentina	1	F38681
*Tanacetum parthenium* (L.) Sch. Bip.	Rainha-das-ervas	5	L1219
*Tanacetum vulgare* L.	Catinga-de-mulata, Erva-mulata	5	L1135
*Taraxacum officinale* F.H. Wigg.	Dente-de-leão	1	L1146
*Vernonia condensata* Baker	Figatil, figatil-índio, Boldo-chileno	4	L1159
*Vernonia scorpioides* (Lam.) Pers.	Mata-pasto, São-simão	4	L1194
*Vernonia polyanthes* Less.	Assa-peixe	2	L1116
Basellaceae			
*Anredera cordifolia* (Tem.) Steenis	Macarrão	2	L1185
Bignoniaceae			
*Jacaranda micrantha* Cham.	Caroba, baratimã	1	L1132
*Jacaranda puberula* Cham.	Caroba-roxa	1	NI
*Macfadyena unguis-cati* (L.) A.H. Gentry	Unha-de-gato	1	NI
*Tabebuia pulcherrima *Sandwith	Ipê-roxo	2	L1175
Boraginaceae			
*Cordia verbenacea* DC.	Baleeira	5	L1119
*Symphytum officinale* L.	Confrei	7	L1144
Brassicaceae			
*Brassica oleracea* L.	Couve	1	
*Coronopus didymus* (L.) Sm.	Menstruz, manstrucho, menstruz-sementinha, menstruzo	14	L1198
*Lepidium aletes* J. F. Macbr.	Menstruzo-vassorinha, pinheiro-santo	1	L1126
*Nasturtium officinale* R. Br.	Agrião	5	IC
Bromeliaceae			
*Tillandsia* sp.	Gravatá-laranjeira	1	L1166
Cactaceae			
*Opuntia* sp.	Arumbeva, palma	1	NI
*Pereskia aculeata* Mill.	Amém	1	L1112
*Rhipsalis baccifera* (J. S. Muell.) Stearn	Erva-de-passarinho	1	L1220
Caricaceae			
*Carica papaya* L.	Mamão, mamão-macho	2	IC
Celastraceae			
*Maytenus aquifolium* Chodat	Espinheira-santa	2	L1155
Convolvulaceae			
*Ipomoea batatas* (L.) Lam.	Batata-doce	1	IC
Clusiaceae			
*Garcinia gardneriana* (Planch. & Triana) Zappi	bacupari	3	L1118
Commelinaceae			
*Commelina *cf. *benghalensis* L.	Capoerage, trapoeiraba, mato-que-o-grilo-dorme	1	L1230
*Dichorisandra thyrsiflora* J. C. Mikan	Cana-do-brejo-da-roxa	3	L1127
*Tradescantia zebrina* Heynh.	Trapoeiraba, ondas-do-mar	2	L1230
Costaceae			
*Costus *sp.	Cana-do-brejo	5	
*Costus spicatus* (Jacq.) Sw.	Cana-do-brejo	1	L1226
Crassulaceae			
*Bryophyllum pinnatum* (Lam.) Oken	Fortuna	4	L1161
Cucurbitaceae			
*Sechium edule* (Jacq.) Sw.	Chuchu, chuchu-amarelo	8	L1140
*Cucurbita *sp.	Abóbora	2	IC
Cyperaceae			
*Bulbostylis capillaris* (L.) Kunth ex C. B. Clarke	Cabelo-de-porco	1	F38673
*Scirpus *sp.	Piri	1	NI
Dioscoreaceae			
*Dioscorea altissima* Lam.	Salsa-parrilha	9	L1223
*Dioscorea laxiflora* Mart. ex Griseb.	Taiua	1	L1228
Equisetaceae			
*Equisetum giganteum* L.	Cavalinha, rabo-de-lagarto, Cana-cavalinha	8	L1136
Euphorbiaceae			
*Aleurites fordii* Hemsl.	Anozeiro, anoz	1	L1115
*Jatropha multifida* L.	Mercúrio-da-horta, Cura-corte, Metiolate	3	L1200
*Manihot esculenta* Crantz	Aipim, mandioca	2	IC
*Ricinus communis* L.	Mamoneira, carrapateira	2	L1190
Fabaceae			
*Bauhinia forficata* Link	Pata-de-vaca	1	IC
*Bauhinia microstachya* (Raddi) J. F. Macbr.	Pata-de-vaca	5	L1206
*Bauhinia* sp.	Pata-de-vaca	3	NI
*Cajanus cajan* (L.) Huth	Feijão-andu, feijão-guandu	4	L1157
*Indigofera suffruticosa* Mill.	Erva-de-anil	2	L1147
*Mucuna urens* (L.) Medik.	Olho-de-boi, corronha, curriancho	1	L1214
*Senna corymbosa* (Lam.) H. S. Irwin & Barneby	Fidigoso-bravo	1	F38675
*Zollernia ilicifolia* (Brongn.) Vogel	Espinheira-santa	2	L1156
Geraniaceae			
*Pelargonium* sp.	Malva-cheirosa, malva-simples	2	L1186
Labiaceae			
*Leonotis nepetifolia* (L.) R. Br.	Cordão-de-são-francisco, cordão santo	3	L1145
Lamiaceae			
*Hyptis *sp.	Mata-vilida, pau-de-negro	1	L1196
*Hyptis suaveolens* (L.) Poit.	Erva-cidreira	11	L1151
*Lavandula angustifolia* Mill.	Alfazema	5	L1110
*Mentha pulegium* L.	Poejo	5	L1211
*Mentha* sp1. L.	Hortelã, hortelã branca, hortelã-roxa	23	L1172
*Mentha* sp2.L.	Menta, vic	2	L1233
*Mentha* sp3. L.	Alevante, elevante, levante	3	L1180
	Manjericão-de-folha-mais-escura	1	L1189
*Ocimum campechianum* Mill.	Erva-doce, anis, alfavaca, são simão	10	L1148
*Origanum vulgare* L.	Orégano	1	L1215
*Plectranthus barbatus* Andrews	Boldo, boldo-de-chile, boldo-do-brasil	9	L1122
*Plectranthus neochilus* Schltr.	Boldo-miúdo	1	L1124
*Rosmarinus officinalis* L.	Alecrim	11	L1108
*Salvia splendens* Sellow ex Wied-Neuw.	Chá-do-reino	1	L1138
*Tetradenia riparia* (Hochst.) Codd	Incenso	3	L1173
*Vitex megapotamica* (Spreng.) Moldenke	tarumã, cinco-folha, nó-de-cachorro	2	L1212
Lauraceae			
*Cinnamomum zeylanicum* Blume	Canela, quina-do-mato	1	L1234
*Laurus nobilis* L.	Loro	8	L1182
*Ocotea odorifera* Rohwer	Canela-sassafraz	5	NI
*Persea americana* Mill.	Abacate	7	IC
Lythraceae			
*Cuphea carthagenensis* (Jacq.) J. F. Macbr.	Sete-sangria, TACO-de-índio, BOA-noite	6	F38678
Lythraceae			
*Punica granatum* L.	Romã	6	L1221
Malvaceae			
*Gossypium hirsutum* L.	Algodão	2	
*Luehea divaricata* Mart.	Açoita-cavalo	2	L1107
*Malva parviflora* L.	Malva-de-dente	4	L1187
*Malva* sp.	Malva	9	
*Malvastrum coromandelianum* (L.) Garcke	Guaxuma	1	L1169
*Bombacopsis glabra* (Pasq.) A. Robyns	Castanha	1	L1134
*Triumfetta* sp.	Carrapicho	2	L1133
Meliaceae			
Melia azedarach L.	Cinamomo	1	NI
Myristicaceae			
*Myristica fragrans* Houtt.	Noz-noscada	1	NI
Moraceae			
*Ficus* sp.	Figueira-branca	1	NI
*Ficus pumila* L.	Folha-de-hera	1	L1171
*Morus nigra* L.	Amora	3	L1113
Musaceae			
*Musa* sp.	Banana	2	
Myrtaceae			
*Eucalyptus citriodora* Hook.	Eucalipto-lima	3	IC
*Eugenia uniflora* L.	Pitanga	7	L1210
*Psidium cattleyanum* (Mart. ex O. Berg) Kiaersk.	Araçá	6	IC
*Psidium guajava* L.	Goiaba	6	IC
*Syzygium cumini* (L.) Skeels	Gibolão, cerejeira, Jambolão	2	L1165
Nyctaginaceae			
*Boerhavia diffusa* L.	Erva-tostão, erva-tristão, erva-tustão	3	F38671
Onagraceae			
*Oenothera mollissima* L.	Miliã	1	L1201
Oxalidaceae			
*Averrhoa carambola* L.	Carambola	1	NI
*Oxalis* spp. L.	Trevo	1	NI
Passifloraceae			
*Passiflora edulis* Sims	Maracujá	7	L1191
Phyllanthaceae			
*Phyllanthus tenellus* Roxb.	Quebra-pedra	10	L1216
Phytolacaceae			
*Petiveria alliacea* L.	Guiné	4	IC
Piperaceae			
*Ottonia martiana* Miq.	Jaborandin	1	L1176
*Piper* sp.	Pariparoba	1	L1204
*Piper *cf.* umbellatum* L.	Pariri	4	L1203
Plantaginaceae			
*Plantago australis* Lam.	Tansagem, tansagem-nativa, carssá	3	F38672
*Plantago major* L.	Tansagem	2	L1229
*Plantago* sp.	Tansagem	11	NI
Poaceae			
*Coix lacryma-jobi* L.	Lágrima-de-nossa-senhora	1	NI
*Cymbopogon citratus* (DC.) Stapf	Cana-cidreira, capim-cidrão, capim-santo	12	L1129
*Cymbopogon winterianus* Jowitt ex Bor	Citronela	1	NI
*Eleusine tristachya* (Lam.) Lam.	Capim-pé-de-galinha	1	F38680
*Melinis repens* (Willd.) Zizka	Capim-graxa	1	NI
*Saccharum officinarum* L.	Cana, cana-de-açucar	4	IC
*Zea mays* L.	Milho	2	IC
Polypodiaceae			
*Microgramma vacciniifolia* (Langsd. & Fisch.) Copel.	Cipó-cabeludo	1	L1142
Polygalaceae			
*Polygala cyparissias* A. St.-Hil. & Moq.	Gelol	3	IC
Proteaceae			
*Roupala *cf.* brasiliensis* Klotzsch	Carvalho	1	NI
Pteridaceae			
*Adiantum *cf.* raddianum* C. Presl	Avenca	3	L1117
Rosaceae			
*Eriobotrya japonica* (Thunb.) Lindl.	Ameixa	2	L1111
*Rosa *spp.l.	Rosa-branca, rosa-branca-verdadeira, rosa-vermelha, rosas	5	IC
*Rubus *sp.	Amora-do-mato	1	NI
Rubiaceae			
*Coffea arabica* L.	Café	2	L1125
*Diodia radula* (Willd. ex Roem. & Schult.) Cham. & Schltdl.	Erva-lagarto	3	L1152
Rutaceae			
*Citrus limon* (L.) Osbeck	Limão	3	L1181
*Citrus reticulata* Blanco	Laranja-crava	2	L1179
*Citrus sinensis* (L.) Osbeck	Laranja, laranja-azeda, laranja-bruta	14	L1178
*Ruta graveolens* L.	Arruda	5	IC
Salicaceae			
*Casearia sylvestris* Sw.	Chá-de-bugre	1	L1137
Sapindaceae			
*Paullinia cupana* Kunth	Guaraná	1	L1170
Simaroubaceae			
*Picrasma crenata* Engl. In Engl. & Prantl	Pau-amargo, pau-de-velha, pau-pra-tudo	4	L1207
Solanaceae			
*Datura suaveolens* Humb. & Bonpl. ex Willd.	Buzina	1	IC
*Solanum lycopersicum* L.	Tomate-miúdo	1	IC
*Solanum *cf.* paniculatum* L.	Jurubeba	4	L1177
Solanum tuberosum L.	Batata, batata-inglesa		IC
Theaceae			
*Thea sinensis* L.	Chá-preto	1	NI
Tropaeolaceae			
*Tropaeolum majus* L.	Chaga-de-cristo, capuchinha	1	IC
Urticaceae			
*Cecropia* sp.	Embaúva	1	NI
*Parietaria* sp.	Parietária	1	L1202
*Urera baccifera* (L.) Gaudich. ex Wedd.	Urtigão	1	L1232
Verbenaceae			
*Aloysia gratissima* (Gillies & Hook.) Tronc.	Erva-santa, erva-de-santa-maria, folha-santa, erva-das-dores	3	L1153
*Aloysia triphylla* Royle	Cidrão	8	L1141
*Lantana camara* L.	Bem-me-quer, calenda, mal-me-quer	5	L1121
*Lippia alba* (Mill.) N.E. Br. ex Britton & P. Wilson	Melissa, erva-melissa, salvia	13	L1197
*Stachytarpheta cayennensis* (Rich.) Vahl	Gervão, gervão-branco, gervão-roxo, zervão-roxo	6	L1164
Violaceae			
*Viola odorata* L.	Violeta-roxa	2	L1231
Vitaceae			
*Cissus sicyoides* L.	Insulina	3	L1174
*Vitis vinifera* L.	Uva	1	IC
Xanthorrhoeaceae			
*Aloe* sp1.	Babosa-de-folha-larga	1	NI
*Aloe* sp2.	Babosa	8	NI
Zingiberaceae			
*Hedychium coronarium* J. König	Noz-noscada-do-brejo	1	L1213

*¹*In the not identified (NI) category the species collected in the field, but that were not possible to identify botanically, and species not collected were included, however, some of these were identified based on the common names.
